# BACH1: A Potential Predictor of Survival in Early-Stage Lung Adenocarcinoma

**DOI:** 10.1155/2022/3921095

**Published:** 2022-01-06

**Authors:** Jin Zhou, Zheming Liu, Huibo Zhang, Tianyu Lei, Jiahui Liu, Yaqi Zhao, Yi Yao, Qibin Song

**Affiliations:** ^1^Cancer Center, Renmin Hospital of Wuhan University, Wuhan, China; ^2^Anesthesiology Department, Renmin Hospital of Wuhan University, Wuhan, China

## Abstract

**Purpose:**

Recent researches showed the vital role of BACH1 in promoting the metastasis of lung cancer. We aimed to explore the value of BACH1 in predicting the overall survival (OS) of early-stage (stages I-II) lung adenocarcinoma. *Patients and Methods*. Lung adenocarcinoma cases were screened from the Cancer Genome Atlas (TCGA) database. Functional enrichment analysis was performed to obtain the biological mechanisms of BACH1. Gene set enrichment analysis (GSEA) was performed to identify the difference of biological pathways between high- and low-BACH1 groups. Univariate and multivariate COX regression analysis had been used to screen prognostic factors, which were used to establish the BACH1 expression-based prognostic model in the TCGA dataset. The C-index and time-dependent AUC curve were used to evaluate predictive power of the model. External validation of prognostic value was performed in two independent datasets from Gene Expression Omnibus (GEO). Decision analysis curve was finally used to evaluate clinical usefulness of the BACH1-based model beyond pathologic stage alone.

**Results:**

BACH1 was an independent prognostic factor for lung adenocarcinoma. High-expression BACH1 cases had worse OS. BACH1-based prognostic model showed an ideal C-index and *t*-AUC and validated by two GEO datasets, independently. More importantly, the BACH1-based model indicated positive clinical applicability by DCA curves.

**Conclusion:**

Our research confirmed that BACH1 was an important predictor of prognosis in early-stage lung adenocarcinoma. The higher the expression of BACH1, the worse OS of the patients.

## 1. Introduction

Lung cancer (LC) is one of the malignant tumors that threatens the health and life of human being [1]. In the past 50 years, many countries have reported a significant increase in lung cancer morbidity and mortality [2], which accounts for the first place in all malignant tumors in male and the second place in female [2, 3]. Among all the pathological phenotypes of LC, non-small-cell lung cancer (NSCLC) presents with the highest morbidity, especially lung adenocarcinoma [4].

Transcription factors are proteins that bind to DNA regulatory sequences to modulate gene transcription, which may result in alteration in gene transcription, protein synthesis, and cellular function. Transcriptional activators promote gene transcription and repressors inhibit that of Reference [5]. BTB and CNC homology 1 (BACH1) belongs to the bZIP [6] transcription factor family [7]. BACH1 mRNA is highly expressed in subsets of monocytes, macrophages, neutrophils, and dendritic cells, which are abundant in the tumor microenvironment (TME) [5]. As these immune cells orchestrate nearly all of the proliferation, differentiation, and metastasis in the development of solid tumor, the TME system has been recognized as the most promising antitumor therapy [8].

As it is reported recently, the reactive oxygen system (ROS) has been defined as another important factor in the tumor tissue homeostasis and cellular differentiation and metastasis. Antioxidant transcription factor NRF2 are abundant in cancer, which suggested that increased antioxidant defense contributes to the tumor progression. ROS plays a vital role in the homeostasis in normal or tumor tissue and hints its contradictory and complex role in the TME system [9]. Lee et al. found that heme can inhibit the development of triple-negative breast cancer (TNBC) through the suppression of activation of BACH1, which can negatively modulate the gene expression of electron transport chain (ETC) in mitochondria [10]. Lignitto et al. [11] and Wiel et al. [12] groups reported NRF2 activation in KrasG12D; p53flox/flox lung tumor mouse model can indirectly promote the stability of BACH1 via the inhibition of heme and at last induce the metastasis of lung cancer, which may be contributed by antioxidant treatment.

The above studies indicated that the upregulation of BACH1 promoted lung cancer metastasis. However, there is no direct evidence of BACH1 expression in relation to the prognosis of early-stage lung cancer.

We utilized bioinformatics approach such as the Cancer Genome Atlas (TCGA) and Gene Expression Omnibus (GEO) database to explore the role of BACH1 expression in the prognosis of early-stage lung adenocarcinoma. In this study, we established a BACH1-related prognostic model to predict overall survival (OS) of early-stage lung adenocarcinoma. In addition, bioinformatics analyses were performed to explore the biological processes and possible cell signal pathways underlying the prognosis.

## 2. Materials and Methods

### 2.1. Data Acquisition

The gene expression data and corresponding clinical information of lung adenocarcinoma were screened from the Cancer Genome Atlas (TCGA) website (https://portal.gdc.cancer.gov/repository) (up to May 17, 2020). 515 cases with RNA-sequencing data and clinical information were initially downloaded. The gene expression profiles were normalized by variance stabilizing transformation (VST) using DESeq2 R package. 134 cases with less than 30 days of following-up time and 75 cases with unknown clinical stage or stages III-IV were excluded; eventually, 306 cases were enrolled for subsequent analysis.

### 2.2. Functional Enrichment Analysis

Spearman correlation analysis was performed between the expression of BACH1 and other encoding genes, and then genes with *P* value less than 0.05 and the highest correlation coefficient (>0.3) were selected. Gene Ontology (GO) analysis was performed using the clusterProfiler R package [13] to evaluate the BACH1-related biological process (BP), cellular compartment (CC), and molecular function (MF). Kyoto Encyclopedia of Genes and Genomes (KEGG) analysis was conducted to further evaluate potential biological signal pathways related to BACH1 expression. We visualized significant processes and pathways using the function of clusterProfiler R package.

### 2.3. Gene Set Enrichment Analysis

Gene set enrichment analysis (GSEA) was performed to identify the difference of biological pathways and corresponding genes between lung adenocarcinoma cases with the high- and low-BACH1 groups, in order to further evaluate the potential mechanism of the underlying involvement of BACH1 in lung adenocarcinoma prognosis. An annotated gene set file (h.all.v7.1.entrez.gmt) was selected as reference. The threshold was set at *P* < 0.05.

### 2.4. Construction of BACH1 Expression-Based Prognostic Model

BACH1 expression data and clinical information were integrated to analyze the relationship between BACH1 and OS. Univariate and multivariate COX regression analyses were conducted to select prognostic factors. Then, variables that achieved significance at *P* < 0.05 after the multivariable analysis were screened to establish the nomogram model. Concordance index (C-index) was used to quantify the predictive accuracy of the model. C-index ranges from 0.5, which means a random chance, to 1.0, which indicates a perfect ability of correct prediction. The calibration plot was performed to compare actual and predicted probability of 3- and 5-year OS. Then, the reliability of the model was verified by means of the time-dependent area under ROC curves (*t*-AUC). A *t*-AUC value above 0.7 suggests that a reasonable prediction model has been constructed.

### 2.5. External Validation of Prognostic Model

We systematically searched for gene expression datasets of early-stage lung adenocarcinoma that were published and available in Gene Expression Omnibus (GEO) website (https://ncbi.nlm.nih.gov/geo). We finally selected two cohorts of samples in GEO databases (GSE13213 and GSE72094) as external validation cohorts to further validate the value of BACH1 expression-based prognostic model.

### 2.6. Risk Group Stratification Based on the Nomogram and Clinical Usefulness

Log-rank statistics was used to make a risk group stratification according to the total risk scores based on the nomogram, in order to illustrate the independent discrimination ability of BACH1-based model beyond BACH1 alone. Decision curve analysis (DCA) [14, 15] was finally used to evaluate clinical usefulness of BACH1-based model beyond pathologic stage alone.

All analyses were conducted in R software (version 3.6.1). The value of *P* < 0.05 was statistically significant.

## 3. Results and Discussion

### 3.1. Characteristics of Cases in TCGA Dataset

A total of 276 cases of TCGA cohort with both clinical and gene expression data were enrolled in the present study (Table 1). The median follow-up time was 20 months, and median age was 66 years old. 44.1% cases were male. The pathologic stage included 215 (70.3%) with stage I and 91 (29.7%) with stage II. Most cases (254, 83.0%) had smoking history. 48 of 204 (23.5%) cases had lymph node metastases (pelvic and para-aortic). Cases with high- and low-expression BACH1 accounted for 33.7% (103) and 66.3% (203), respectively.

### 3.2. Functional Enrichment Analysis

**Figure 1:**
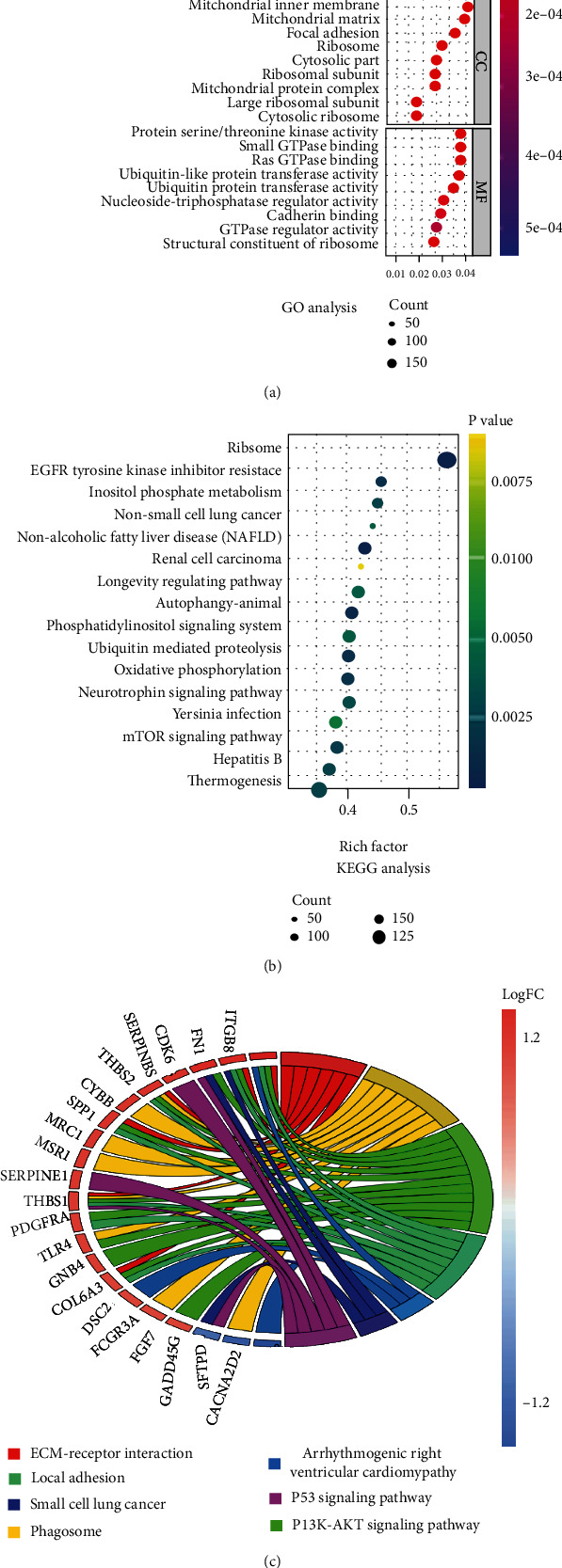
GO and KEGG analysis of BACH1 in the training set (TCGA dataset). (a) GO analysis of the BACH1-related BP, CC, and MF. (b) KEGG analysis of potential biological signal pathways related to BACH1 expression. (c) Circular plot of the KEGG pathways enriched for BACH1 expression-related genes.

GO and KEGG analysis was performed to obtain a novel understanding of biological mechanisms of BACH1. 5000 genes that highly associated with BACH1 (correlation coefficient > 0.3 and *P* < 0.05) were extracted and subjected to GO and KEGG analyses. Genes related to BACH1 expression were mainly enriched in BP column of “protein targeting”, “nuclear-transformed mRNA catabolic process”, “protein localization to endoplasmic reticulum”, “protein targeting to endoplasmic reticulum”, “cotranslational protein targeting to membrane” terms, and in CC column of “mitochondrial inner membrane”, “mitochondrial matrix”, “focal adhesion”, “ribosome”, “cytosolic part” terms, and in MF column of “protein serine/threonine kinase activity”, “small GTPase binding”, “RAS GTPase binding”, “ubiquitin-protein transferase activity”, “nucleoside-triphosphatase regulator activity”, “cadherin binding”, “GTPase regulator activity”, and “structural constituent of ribosome” terms according to the GO analysis (Figure 1(a)), as well as “ribosome”, “EGFR tyrosine kinase inhibitor resistance”, “inositol phosphate metabolism”, “non-small-cell lung cancer”, “autophagy”, “oxidative phosphorylation”, and “mTOR signaling pathway” according to KEGG analysis (Figure 1(b)). Furtherly, we screened out 200 genes with the most significant correlation with BACH1 expression to construct circular plot of KEGG and found that the PI3K-Akt signaling pathway, which was known to be a signaling pathway closely related to the occurrence and development of tumors, was significantly activated (Figure 1(c)) in the high-expression group of BACH1.

### 3.3. Potential Mechanism Underlying the Role of BACH1 Affecting Prognosis

GSEA was performed to identify the difference of biological pathways and corresponding genes between 103 high- and 203 low-expression BACH1 cases. 28 biological processes were significantly enriched (Table 2), 21 activated, and 7 suppressed cell signal pathways.

**Figure 2:**
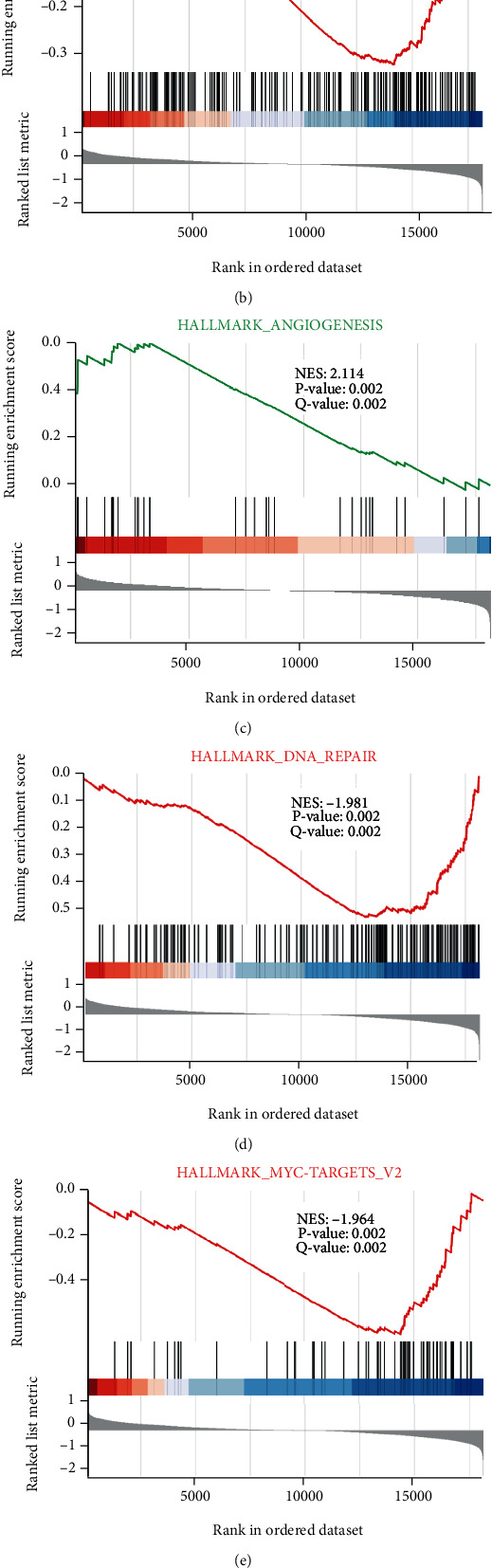
GSEA analysis of BACH1 in the training set (TCGA dataset). Significant enrichment of the BACH1-related signaling pathways in the high-expression BACH1 group compared with that in the low-expression BACH1 group.

We selected the most significantly enriched pathways based on normalized enrichment score (NES) in BACH1 high-expression phenotype. The results revealed that OXIDATIVE_PHOSPHORYLATION (NES = −2.575, *P* = 0.002), MYC_TARGETS_V1 (NES = −1.448, *P* = 0.002), DNA_REPAIR (NES = −1.981, *P* = 0.002), and MYC_TARGETS_V2 (NES = −1.964, *P* = 0.002) pathways were differentially suppressed. ANGIOGENESIS (NES = 2.114, *P* = 0.002), IL6_JAK_STAT3_SIGNALING (NES = 1.908, *P* = 0.002), and TGF_BETA_SIGNALING (NES = 1.860, *P* = 0.002) pathways were differentially activated (Figure 2).

## 4. Development and Validation of BACH1-Based Prognostic Model

**Figure 3:**
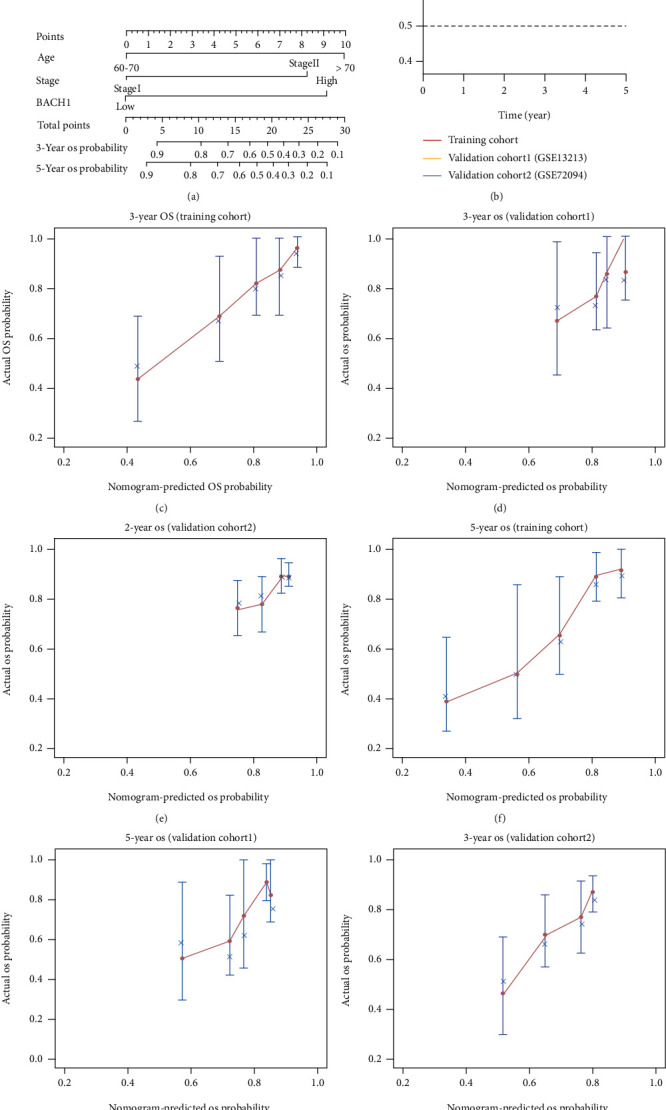
Nomogram construction and evaluation. (a) A constructed nomogram for risk prediction of OS. (b) T-AUC of the nomogram model in the TCGA dataset and validation cohort (GSE13213, GSE72094). (c) Calibration curves of the 3-year OS in the TCGA dataset. (d) Calibration curves of the 3-year OS in the validation cohort 1 (GSE13213). (e) Calibration curves of the 2-year OS in the validation cohort 2 (GSE72094). (f) Calibration curves of the 5-year OS in the TCGA dataset. (g) Calibration curves of the 5-year OS in the validation cohort 1 (GSE13213). (h) Calibration curves of the 3-year OS in the validation cohort 2 (GSE72094).

Univariate and multivariate COX regression analyses were used to select risk factors. According to Cox regression analysis (Table 3), age (*P* < 0.001), pathologic stage (*P* < 0.001), and BACH1 expression (*P* < 0.001) were significantly independent prognostic factors and were incorporated to establish the nomogram model (Figure 3(a)).

The predictive ability of the model was then evaluated in TCGA dataset and independently validated in the validation cohort of GSE13213 and GSE72094.

The C-index of the model was 0.782 (95% CI [0.752, 0.812]) in TCGA dataset while 0.648 (95% CI [0.596, 0.700]) in GSE13213 cohort and 0.632 (95% CI [0.595, 0.669]) in GSE72094 cohort.  Figure 3(b) shows that *t*-AUC value was above 0.6 for the prediction of deterioration risk within 5 years both in TCGA dataset and validation cohort, indicating that a stable prognostic model was established.

Furthermore, the calibration curves of the model showed high consistencies between predicted and observed 3- and 5-year OS probability in TCGA dataset and GSE13213 cohort (Figures 3(c), 3(d), 3(f), and 3(g)) and 2- and 3-year OS probability in GSE72094 cohort. Thus, the model showed considerably discriminative and calibrating abilities.

## 5. Risk Stratification Based on the Nomogram

**Figure 4:**
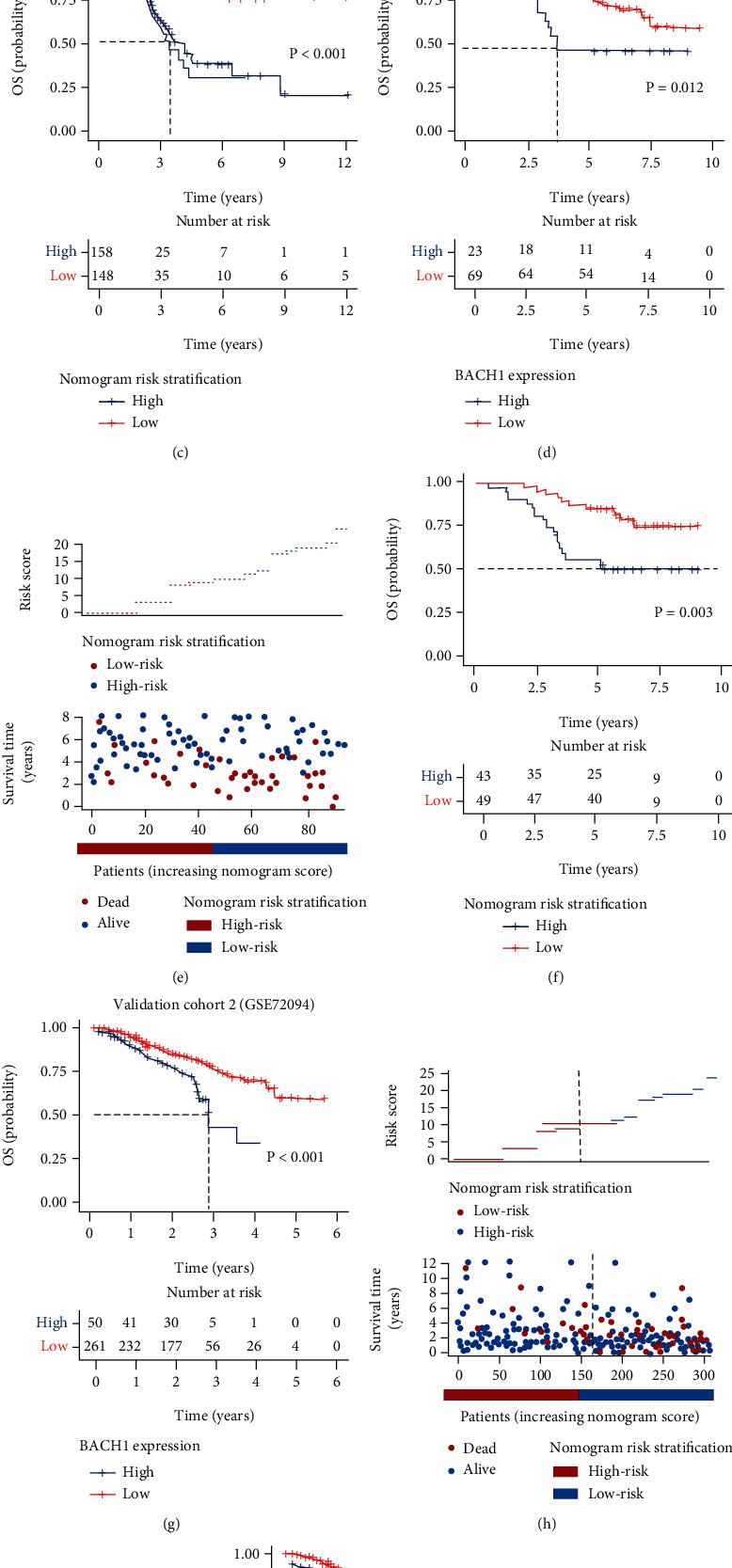
Analyses of BACH1 expression on OS at different risks in lung adenocarcinoma cases. (a, d, g) Impact of BACH1 expression on OS in the TCGA dataset, validation cohort 1 (GSE13213), and validation cohort 2 (GSE72094). (b, e, h) Cut-off point selection for risk stratification according to nomogram scores in the TCGA dataset, validation cohort 1 (GSE13213), and validation cohort 2 (GSE72094). (c, f, i) Kaplan-Meier curves of OS at different risks in the training and validation cohort (before PSM). (f, g) Kaplan-Meier curves of C-DFS at different risks based on nomogram scores in the TCGA dataset, validation cohort 1 (GSE13213), and validation cohort 2 (GSE72094).

High-expression BACH1 cases had worse OS according to Kaplan-Meier curves (TCGA dataset, *P* < 0.001; GSE13213 cohort, *P* = 0.012; GSE72094 cohort, *P* = 0.003) (Figures 4(a), 4(d), and 4(g)). Risk stratification was made based on the nomogram. The total point of each case was counted based on the score of each variable. Cases were grouped into two risk groups according to total points by utilizing X-tile software: low-risk (total points < 10) and high-risk (total points ≥ 10) group. The risk plot showed that the deaths occurred more frequently in the high-risk group in both TCGA and validation cohort (Figures 4(b), 4(e), and 4(h)). The Kaplan-Meier curves also presented the significant discrimination among two risk groups both in TCGA (*P* < 0.001) and validation cohort (GSE132123, *P* = 0.003; GSE72094, *P* = 0.002).

### 5.1. Clinical Usefulness

**Figure 5:**
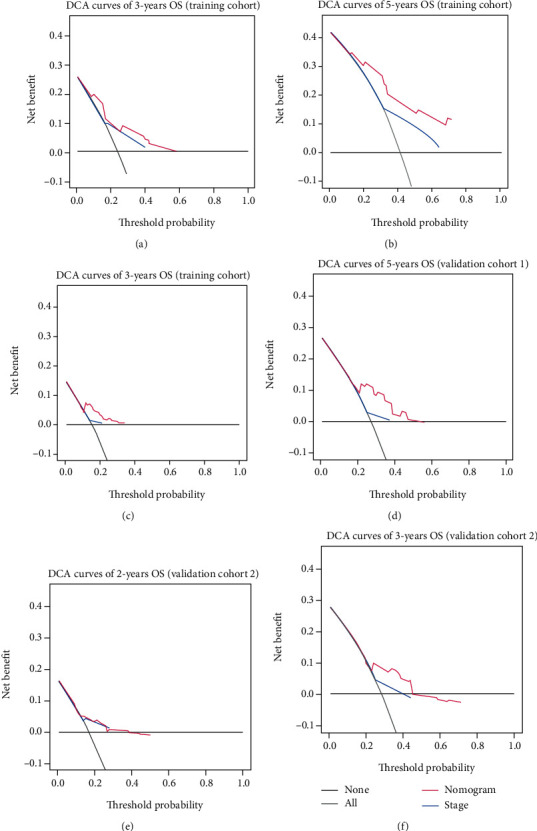
DCA curves of nomogram model compared with pathologic stage. (a, b) DCA curves of 3- and 5-year in the TCGA dataset. (c, d) DCA curves of 3- and 5-year in the validation cohort 1 (GSE13213). (e, f) DCA curves of 2- and 3-year in the validation cohort 2 (GSE72094).

DCA was applied to evaluate the clinical usefulness of the model by quantifying the net benefit at different threshold probabilities compared with stage systems (Figure 5). The model showed more net benefits than stage systems across a wider range of threshold probabilities both in TCGA dataset and validation cohort.

## 6. Discussion

In this pioneering study, we confirmed that BACH1 was an important prognostic factor for early-stage lung adenocarcinoma by establishing a BACH1-based prognostic model that incorporated BACH1 expression and clinical characteristics. The prognostic model was evaluated by a variety of statistical indicators and validated by independent datasets and proved to be accurate. More importantly, the BACH1-based model indicated positive clinical applicability by DCA curves.

Countless molecular factors contribute to the proliferation and metastasis of cancer, which meant its thousand years living as one of the most malignant diseases with us human beings. BACH1 on behalf of bad prognosis gene has been elucidated by the presentation of its biofunction and molecular mechanism. Lignitto et al. [11] and Wiel et al. [12] groups reported that NRF2 activation can indirectly promote the stability of BACH1 and at last induce the metastasis of lung cancer; however, no research had ever illustrated whether BACH1 affects the prognosis of early-stage lung cancer. We firstly confirmed the vital role of BACH1 in the prognosis of early-stage lung cancer.

With the highest mortality, lung adenocarcinoma also shows more gene mutation and leads to its wide variety of treatments [16, 17]. BACH1-associated gene enrichment suggested the top ones that connected with its biofunction. As a transcription factor, it apparently participated frequently in protein expression and biosystem of protein expression associated process. The protein targeting enrichment may give us a new view to tumor target therapy. BACH1 also participates in viral gene transcription and expression, which hints us its connection with some viral-induced tumor, for example, cervical cancer [18].

Lee et al. reported that BACH1 affected the transcription of electron transport chain (ETC) genes [10], which mainly functioned in the mitochondria. Our big data analysis also yielded similar results, which suggested that BACH1 mainly play vital role in the biological activity in mitochondria. It inhibits the ETC gene transcription and leads to the more available independence from mitochondrial aerobic respiration. What is interested is that BACH1 may contribute to metastasis via the focal adhesion kinase (FAK), which is an important mediator of cell proliferation, differentiation, and migration [19]. Malignant metastasis normally comes down to the ECM or cellular permeation process [20, 21]; it is not surprising that FAK participates in that, which has also been confirmed by studies from both mouse model and human patients. From the cellular view, BACH1 obviously activates the serine/threonine kinase, which always binds to the transforming growth factor-*β*s (TGF-*β*s). Its activation has been estimated as a vital promoter in neoplasia. Another potential tumor-promoting effect may be its binding to the small GTPase, which contains five subfamily members: Ras, Rho, Rab, Sarl/Arf, and Ran. Among them, Ras plays a vital role in the human neoplasia. They are signaling nodes that are activated in response to a variety of extracellular stimuli. Activated Ras combines with various effectors with different catalytic activities to regulate cytoplasmic signal network, so as to control gene expression, cell proliferation, differentiation, and growth.

KEGG analysis comprehensively recapitulates the bioinformation from both macroscopic and microscopic views, which showed us an excellent data that BACH1 may be an important prognosis factor to the NSCLC. Some NSCLC patients, who burden the mutation of epidermal growth factor receptor (EGFR), have pointers to the target therapy [22] and may also have poor therapeutic effect due to high BACH1 expression.

Besides, the PI3K-Akt signaling pathway, which was known to be a signaling pathway closely related to the occurrence and development of tumors, was also significantly activated in the high BACH1 expression cases. GSEA analysis also showed suppressed oxidative phosphorylation and DNA repair pathway and activated oncogenic pathways such as angiogenesis, IL-6/JAK/STAT3, and TGF-*β* signaling pathways in the high-expression group of BACH1.

ROS is a well-known cancer-related system, which could be generated by neutrophils, macrophages, and even tumor cell itself. Due to this complex and contradictory system in the neoplasia [23], cancer cells engage a relative safe environment to survive and proliferate, which we called TME (tumor microenvironment). Through inhibiting BACH1, ROS prevents the malignant proliferation and metastasis; however, BACH1-activated FAK [24] may help the cells to attach to extracellular matrix, thus contributes to the oxidative environment in the solid tumor, which in turn can help BACH1 restrain the ETC gene expression. At last, these factors help to construct a neoplasia-fitted hypoxia microenvironment and promote the proceeding of tumor proliferation. BACH1-enriched immune cells can drive immune storm alone or cooperate together. For example, neutrophils promote tumorigenesis via the release of ROS, which contributes to DNA damage [25]. Animal experiment that conducted in zebrafish showed the cooperation of macrophages and neutrophils in neoplasia, in which macrophages can attract neutrophils through ROS-Src family kinase signaling, which hints the important recruitment role of TME to immune cells how macrophages modulate the attachment of immune cells [23].

## 7. Conclusions

In conclusion, our study confirmed the vital role of BACH1 in the prognosis of early-stage lung adenocarcinoma. The orchestration of a complex cell signal network that affects the proliferation and invasion of cancer destines BACH1 to be a promising predictor of the prognosis of NSCLC and a new potential cancer target.

## Figures and Tables

**Table 1:** Demographic and clinical characteristics of early-stage lung adenocarcinoma cases.

Characteristics	Training cohort (TCGA)		Validation cohort 1 (GSE13213)		Validation cohort 2 (GSE72094)	
	No. of patients	Percent (%)	No. of patients	Percent (%)	No. of patients	Percent (%)
Total no.	306		92		311	
Age						
<60	85	27.8	32	34.8	45	14.5
60-70	126	41.2	18	19.6	116	37.3
>70	95	31.0	42	45.7	150	48.2
Gender						
Male	135	44.1	50	54.3	141	45.3
Female	171	55.9	42	45.7	170	54.7
Pathologic stage						
I	215	70.3	79	85.9	246	79.1
II	91	29.7	13	14.1	65	20.9
Laterality						
Left	119	38.9	—	—	—	—
Right	183	59.8	—	—	—	—
Unknown	4	1.3	—	—	—	—
Smoking history						
Yes	254	83.0	47	51.1	236	75.9
No	52	17.0	45	48.9	75	24.1
BACH1 expression						
High	103	33.7	23	33.3	50	19.2
Low	203	66.3	69	66.7	261	80.8

**Table 2:** Gene sets enriched in high-expression BACH1 cases.

Gene set name/signal pathway	NES	*P* value	*Q* value
HALLMARK_OXIDATIVE_PHOSPHORYLATION	-2.575	0.002	0.002
HALLMARK_MYC_TARGETS_V1	-1.448	0.002	0.002
HALLMARK_DNA_REPAIR	-1.981	0.002	0.002
HALLMARK_MYC_TARGETS_V2	-1.964	0.002	0.002
HALLMARK_ANGIOGENESIS	2.114	0.002	0.002
HALLMARK_ANDROGEN_RESPONSE	1.783	0.002	0.002
HALLMARK_PROTEIN_SECRETION	1.657	0.002	0.002
HALLMARK_IL6_JAK_STAT3_SIGNALING	1.908	0.002	0.002
HALLMARK_TGF_BETA_SIGNALING	1.860	0.002	0.002
HALLMARK_HYPOXIA	1.559	0.003	0.002
HALLMARK_G2M_CHECKPOINT	2.300	0.003	0.002
HALLMARK_COMPLEMENT	1.915	0.003	0.002
HALLMARK_APOPTOSIS	1.411	0.003	0.002
HALLMARK_E2F_TARGETS	1.589	0.003	0.002
HALLMARK_INTERFERON_GAMMA_RESPONSE	1.884	0.003	0.002
HALLMARK_MITOTIC_SPINDLE	2.151	0.003	0.002
HALLMARK_TNFA_SIGNALING_VIA_NFKB	2.265	0.003	0.002
HALLMARK_APICAL_JUNCTION	1.645	0.003	0.002
HALLMARK_IL2_STAT5_SIGNALING	1.877	0.003	0.002
HALLMARK_KRAS_SIGNALING_UP	2.349	0.003	0.002
HALLMARK_MTORC1_SIGNALING	1.372	0.003	0.002
HALLMARK_EPITHELIAL_MESENCHYMAL_TRANSITION	3.165	0.003	0.002
HALLMARK_INFLAMMATORY_RESPONSE	2.423	0.003	0.002
HALLMARK_PEROXISOME	-1.526	0.010	0.006
HALLMARK_XENOBIOTIC_METABOLISM	-1.393	0.015	0.009
HALLMARK_COAGULATION	1.291	0.024	0.013
HALLMARK_HEDGEHOG_SIGNALING	1.536	0.026	0.014
HALLMARK_REACTIVE_OXYGEN_SPECIES_PATHWAY	-1.481	0.032	0.016

NES: normalized enrichment score; NOM: nominal. Gene sets with *P* value < 0.05 and *Q* value < 0.05 are considered as significant.

**Table 3:** Univariate and multivariate Cox analyses of OS in training cohort.

Characteristics	Univariable analysis			Multivariable analysis		
	HR	95% CI	*P*	HR	95% CI	*P*
Age			0.002			0.001
<60	1.000			1.000		
60-70	0.698	0.313-1.556	0.379	0.674	0.299-1.521	0.342
>70	2.290	1.143-4.587	0.019	2.355	1.145-4.841	0.020
Gender			0.292			
Male	1.000					
Female	1.227	0.695-2.167	0.480			
Pathologic stage			<0.001			<0.001
I	1.000			1.000		
II	2.673	1.516-4.712	0.001	2.819	1.578-5.036	<0.001
Laterality			0.582			
Right	1.000					
Left	1.118	0.632-1.975	0.475			
Unknown	0.991	0.134-7.343	0.993			
Smoking history			0.375			
Yes	1.000					
No	1.195	0.610-2.341	0.603			
BACH1 expression			<0.001			<0.001
High	1.000			1.000		
Low	0.284	0.158-0.511	<0.001	0.315	0.174-0.571	<0.001

## Data Availability

The data supporting this study are from previously reported studies and datasets, which have been cited.
